# The non-glycosylated protein of *Toxocara canis* MUC-1 interacts with proteins of murine macrophages

**DOI:** 10.1371/journal.pntd.0010734

**Published:** 2022-09-02

**Authors:** Rongqiong Zhou, Hongguo Jia, Zhendong Du, Aiyun Jiang, Zhenhui Song, Tao Wang, Aifang Du, Robin B. Gasser, Guangxu Ma

**Affiliations:** 1 College of Veterinary Medicine, Southwest University, Chongqing, China; 2 Immunology Research Center, Medical Research Institute, Southwest University, Chongqing, China; 3 Institute of Preventive Veterinary Medicine, Zhejiang Provincial Key Laboratory of Preventive Veterinary Medicine, College of Animal Sciences, Zhejiang University, Hangzhou, China; 4 Department of Veterinary Biosciences, Melbourne Veterinary School, The University of Melbourne, Parkville, Victoria, Australia; University of Liverpool, UNITED KINGDOM

## Abstract

Toxocariasis is a neglected parasitic disease caused predominantly by larvae of *Toxocara canis*. While this zoonotic disease is of major importance in humans and canids, it can also affect a range of other mammalian hosts. It is known that mucins secreted by larvae play key roles in immune recognition and evasion, but very little is understood about the molecular interactions between host cells and *T*. *canis*. Here, using an integrative approach (affinity pull-down, mass spectrometry, co-immunoprecipitation and bioinformatics), we identified 219 proteins expressed by a murine macrophage cell line (RAW264.7) that interact with prokaryotically-expressed recombinant protein (r*Tc*-MUC-1) representing the mucin *Tc*-MUC-1 present in the surface coat of infective larvae of *T*. *canis*. Protein-protein interactions between r*Tc*-MUC-1 and an actin binding protein CFL1 as well as the fatty acid binding protein FABP5 of RAW264.7 macrophages were also demonstrated in a human embryonic kidney cell line (HEK 293T). By combing predicted structural information on the protein-protein interaction and functional knowledge of the related protein association networks, we inferred roles for *Tc*-MUC-1 protein in the regulation of actin cytoskeletal remodelling, and the migration and phagosome formation of macrophage cells. These molecular interactions now require verification *in vivo*. The experimental approach taken here should be readily applicable to comparative studies of other ascaridoid nematodes (e.g. *T*. *cati*, *Anisakis simplex*, *Ascaris suum* and *Baylisascaris procyonis*) whose larvae undergo tissue migration in accidental hosts, including humans.

## Author summary

Toxocariasis is a neglected parasitic disease of humans caused mainly by larvae of *Toxocara canis*. Given that *T*. *canis* is zoonotic and can infect a range of mammals, there has been substantial interest in host-parasite relationships, with studies showing that *T*. *canis* larvae secrete abundant mucins that effect/modulate immune responses and disease pathogenesis. To improve the understanding of immunomolecular interactions, we investigated the role(s) of the protein component of a mucin (*Tc*-MUC-1) secreted by infective larvae using a well-defined murine macrophage line (RAW264.7). The non-glycosylated recombinant protein of *Tc*-MUC-1 (designated r*Tc*-MUC-1) was shown to interact with at least 219 proteins of RAW264.7 cells, particularly with the actin binding protein (CFL1) and a fatty acid binding protein (FABP5), which are involved in cell migration and phagocytosis, respectively. Based on these findings, we propose that *Tc*-MUC-1 regulates cytoskeletal organisation and signal transduction in host macrophages. It would be interesting to establish, using the integrative experimental approach employed here, whether the role(s) of *Tc*-MUC-1 protein homologues of related ascaridoids are conserved.

## Introduction

Human toxocariasis is a neglected parasitic disease of global importance [1]. It is caused by the larvae of *Toxocara* species, usually transmitted from domestic and stray canids or felids to humans and other mammals via the ingestion of infective eggs from the contaminated environment, food or water, or via infective larvae in undercooked meat [2,3]. Although most people infected with *Toxocara* larvae are asymptomatic, it has been estimated that ~ 1.4 billion people worldwide are exposed to, or infected with, *T*. *canis* and *T*. *cati*, particularly young children and pet owners [4,5]. The four main forms of human toxocariasis include visceral larva migrans (VLM), ocular larva migrans (OLM), neurotoxocariasis (NT) and covert/common toxocariasis (CT), and there is evidence of associations between anti-*Toxocara* seropositivity and asthma, idiopathic Parkinson’s disease and Alzheimer’s disease in humans [6–11]. Most anthelmintic drugs are not effective against larvae in tissues of the human host, and no vaccine is available for the prevention of human toxocariasis.

Apart from humans, eggs containing larvae of *Toxocara* species can infect many mammalian species as paratenic or accidental hosts; in these hosts, larvae that emerge from the eggs in the host gut migrate to various tissues and cause infection or toxocariasis but do not develop to adults in the small intestine [2]. Fundamental investigations of human toxocariasis have relied heavily on the use of rodent (mouse, rat and jird) models [12,13]. The mouse model has been particularly useful to study host-parasite interactions, including the immunobiology of *Toxocara*/toxocariasis [14]. Clearly, a sound understanding of such interactions in the mouse model at the immunomolecular level is key to underpinning toxocariasis research and to developing new interventions.

Over the past seven decades [15,16], our understanding of the immunobiology of *Toxocara* has improved significantly [17–19]. For example, a series of molecules likely associated with *Toxocara* development, infection and parasitism have been discovered by informatic analyses of genomic, transcriptomic and proteomic data sets [20–26] and explored *in vitro* and *in vivo* [20]. Importantly, lectins (also known as TES-32 and -70) and mucins (also known as TES-120) excreted/secreted by *Toxocara* have been inferred to play crucial roles in the escape of larvae from surrounding cells and ensuing tissue inflammation within host animals [20–22]. Specifically, mucins of *T*. *canis* (i.e. *Tc-*MUC-2, -3, -4 and -5) have been reported to induce the secretion of cytokines by mouse splenocytes and to modulate the Toll-like receptor signalling in human THP-1 macrophages, indicating an involvement in the host-parasite interplay [23,24]. However, apart from mainly serological investigations [25–28], little is known about the molecular processes/mechanisms underlying the interactions between *Toxocara* mucins and host immune cells, particularly at a protein-protein level. In order to gain a better understanding of these aspects, we investigated proteins of murine macrophage cells that interact with a recombinant (non-glycosylated) form of *Tc-*MUC-1 (designated r*Tc-*MUC-1) using a pull-down assay coupled to liquid chromatography-tandem mass spectrometry (LC-MS/MS), explored key protein-protein interactions by co-immunoprecipitation, and inferred structural and functional roles of the *Tc-*MUC-1 protein in host-parasite interactions.

## Methods

### Ethics statement

Adult worms of *T*. *canis* were collected from dogs with naturally acquired infection in the Animal Hospital affiliated to the College of Animal Sciences. Collection and experimentation were approved (permit no. 20170177) by the Ethics Committee of Zhejiang University, Hangzhou, China.

### Worms and cell lines

Eggs were collected from the uteri of adult females of *T*. *canis*, incubated in H_2_O for 8 weeks at 25°C and manually hatched with glass beads. The third-stage larvae (L3s) of *T*. *canis* released from the eggs were purified and enriched using the Baermann method [29], snap frozen in liquid nitrogen and stored in -80°C until use. Murine macrophage cells (RAW264.7) and human embryonic kidney cells (HEK 293T) were purchased from Beyotime Biotechnology, Shanghai, and maintained according to the manufacturer’s instructions.

### RNA extraction and cDNA synthesis

Total RNA was extracted from pooled *T*. *canis* L3s (n = 3000) using Trizol reagent (Ambion, Thermo Fisher Scientific). RNA was reverse-transcribed into the first-strand cDNA using a PrimeScript RT Reagent Kit (Takara Bio, Dalian), according to the manufacturer’s protocol. The synthesised cDNA was stored at -20°C until use.

### Molecular cloning of *Tc-muc-1*

Using the program Primer Premier 5.0 (Premier Biosoft International, Palo Alto, CA), primers (forward: 5´-ATGCACGTCCTTACCGT-3´; reverse: 5´-TTAACAGAAGCCGCACGT-3´) were designed specifically to the mRNA sequence encoding the surface coat glycoprotein TES-120 precursor (*nmuc-1*) of *T*. *canis* (GenBank accession number U39815.1; ref. [30]). Synthesised cDNA template was amplified by PCR in standard buffer containing forward primer and reverse primers (10 pmol/μL each), 2.5 mmol/L Mg^2+^, 2.5 mmol/L of each dNTP and 0.25 U rTaq polymerase (Takara Bio, Dalian) using the following profile: 94°C for 3 min (denaturation), 35 cycles of 94°C for 30 s (denaturation), 55°C for 30 s (annealing) and 72°C for 1 min (extension), then 72°C for 5 min (final extension). PCR products were examined by agarose gel (1.0%) electrophoresis, purified, cloned into the pMD19-T (simple) vector (Takara Bio, Dalian), and competent DH5α *Escherichia coli* (TransGen Biotech, Beijing) transformed. Clones with inserts (n = 3) were selected and insert sequences confirmed by nucleotide acid sequencing (Sangon Biotech, Shanghai). The sequences were compared with those in the NCBI database nucleotide collection (nr/nt) by BLASTn (https://blast.ncbi.nlm.nih.gov/Blast.cgi) and those in SMART, Pfam and signal peptide databases using HMMER [31].

### Prokaryotic expression of r*Tc*-MUC-1

A recombinant form of the *T*. *canis* mucin 1 protein (r*Tc*-MUC-1) fused to a 6× His tag was produced in a prokaryotic expression system. In brief, the *Tc-muc-1* protein-coding sequence was amplified with a primer set containing *Kpn* I and *EcoR* I restriction sites (5’-GGGGTACCATGCACGTCCTTA-3’; 5’-GGAATTCTTAACAGAAGCCGCACGT-3’), and inserted into the pCold TF plasmid (Takara Bio, Dalian) employing the *Kpn* I and *EcoR* I restriction sites. Recombinant plasmids were purified, verified by specific PCR-amplification and competent BL21 (DE3) *E*. *coli* (TransGen Biotech, Beijing) transformed. Following induction with 0.6 mM isopropyl β-d-1-thiogalactopyranoside (IPTG), r*Tc*-MUC-1 was expressed in a culture volume of 250 ml at 37°C for 24 h. Proteins were released from cells by sonication (1 s–on, 3 s–off for 15 min on ice) at 400 W using a Scientz-IID ultrasonic homogenizer (Scientz, China). The sonicates (25 ml each) were centrifuged at 14,000 × g and 4°C for 20 min, and supernatants filtered (0.22 μm aperture) and run through a Ni-NTA column (Sangon Biotech, Shanghai); r*Tc*-MUC-1 was eluted from the column with 250 mM imidazole and subjected to SDS-PAGE analysis using an established protocol [32]. *E*. *coli* transformed with the pCold TF empty plasmid was used as a negative control throughout experimentation.

### Pull-down assay

The RAW264.7 macrophage cell line was cultured in 2 ml Dulbecco’s modification of Eagle Medium (DMEM; Thermo Fisher Scientific) supplemented with 10% (v/v) foetal bovine serum, 100 IU/mL of penicillin, 100 μg/mL of streptomycin and 2.5 μg/mL of amphotericin (Antibiotic–Antimycotic, Thermo Fisher Scientific) and 1% L-glutamine at 37°C and 5% CO_2_. When a confluence of 80% was reached, proteins were extracted from the culture (six wells) by washing RAW264.7 cells twice in phosphate-buffered saline (PBS, pH 7.5), incubation in 1.8 ml non-denaturing lysis buffer (Thermo Fisher Scientific) supplemented with 1 mM phenylmethanesulfonyl fluoride (PMSF; Thermo Fisher Scientific) on ice for 30 min, and centrifugation at 14000 × g (4°C) for 5 min.

A poly-His affinity pull-down method [33] was used to isolate proteins in RAW264.7 macrophage cells that bind r*Tc*-MUC-1. *E*. *coli* expressing r*Tc*-MUC-1 was used to produce the bait protein, which was immobilised on a Ni-NTA column (Sangon Biotech, Shanghai) and washed three times with 2 mL of 10 mM imidazole. The immobilised bait protein was incubated with 1 mL of protein extract from RAW264.7, incubated at 4°C for 4 h and washed three times with 2 mL of 10 mM imidazole. Bait and target proteins were eluted (three times) from the column with 250 mM imidazole. The His-tagged protein expressed by *E*. *coli* containing the empty pCold TF plasmid vector was used as a negative control. Eluted proteins were resolved by SDS-PAGE and stained with silver (Silver Stain for Mass Spectrometry kit, Pierce, Thermo Fisher Scientific).

## LC-MS/MS

An in-gel digestion approach was employed to process the proteins from SDS-PAGE gels. In brief, band slices were reduced with 10 mM (2S,3S)-1,4-bis-sulfanylbutane-2,3-diol, alkylated with 55 mM iodoacetamide and digested with 1 μg trypsin (Pierce, Thermo Fisher Scientific) at 37°C overnight. Peptide desalting was performed using a C18 resin column (Pierce, Thermo Fisher Scientific). Desalted peptides were freeze-dried, resuspended in 15 μL aqueous 3% w/v acetonitrile and 0.1% v/v formic acid, and then subjected to LC-MS/MS using the ekspertTMnanoLC and AB TripleTOF 5600-plus (SCIEX) via a service provider (GeneCreate Biological Engineering Co., Ltd., Wuhan, China). Proteomic data (identifier PXD030007) have been deposited into the PRIDE partner repository of the ProteomeXchange Consortium [34].

Raw mass data were processed using ProteinPilot software (SCIEX) using five search parameters (enzyme of trypsin, Cys alkylation of iodoacetamide, bias correction of true, background correction of true, protein mass of unrestricted) against the *Mus musculus* proteome in the UniProt database. Peptide identification was performed with a confidence of ≥ 95%. Common contaminants (including keratin, antibodies and serum albumin or globulins) were filtered-out prior to the identification of proteins that bind r*Tc*-MUC-1. Identified proteins were annotated based on the UniProt database and Gene Ontology (GO) resources using the OmicsBean data integration analysis platform, and based on the Kyoto Encyclopedia of Genes and Genomes database using the KEGG Orthology-Based Annotation System (KOBAS 3.0) [35]. Protein network analysis was performed using STRING 10.0 software and displayed using Cytoscape 3.4.0 platform [36,37].

### Eukaryotic expression

The protein-coding sequence of *Tc-muc-1* was amplified with primers 5’-CGGGATTCATGCACGTCCTTA-3’ (*Bam*H I restriction site underlined) and 5’-GGAATTCTTAACAGAAGCCGCACGT-3’ (*Eco*R I restriction site underlined), and then inserted into the pcDNA3.1-Flag vector, in order to express a FLAG-tagged *Tc*-MUC-1 fusion protein. HEK 293T cells containing plasmids expressing selected His pull-down proteins of RAW264.7 macrophage cells were prepared. In brief, coding sequences were PCR-amplified using the *Eco*R I- and *Bgl* II-site containing primers (S1 Table), and then inserted into the pCMV-HA vector to produce haemagglutinin (HA)-tagged fusion proteins. Recombinant plasmids were purified using the Qiagen Ultrapure 100 system. HEK 293T cells were transfected with recombinant endotoxin-free plasmids (5 μg) using the ExFect 2000 Transfection Reagent (Vazyme), and cultured in DMEM at 37°C and 5% CO_2_. The culture medium was exchanged with DMEM supplemented with 10% v/v foetal bovine serum 12 h after transfection, and incubated at 37°C and 5% CO_2_ for 36 h.

### Co-IP

The Co-IP assay was employed to confirm the interaction between r*Tc*-MUC-1 and individual partner proteins from RAW264.7 macrophage cells. To extract total proteins, cells transfected with recombinant plasmids were washed with PBS (pH 7.5), suspended in 20 μL NP40 lysis buffer (Beyotime Biotechnology, Shanghai) with 1 mM phenylmethylsulfonyl fluoride (PMSF) on ice for 30 min, and then centrifuged at 14,000 × g (4°C) for 15 min. Protein A superparamagnetic beads (Dynabeads, Thermo Fisher Scientific) were washed twice in PBS (pH 7.5), and then incubated with 3 μL mouse DYKDDDK tag monoclonal antibody (binds to FLAG tag epitope; α-Flag) (Proteintec, Wuhan). Pre-immune serum IgG was used as the negative control. Processed beads were incubated with proteins (50 μL) extracted from HEK 293T cells at 4°C for 4 h, washed three times with PBS (pH 7.5), and mixed with 20 μL PMSF-containing NP40 lysis buffer and 5 μL loading buffer (1.5% Tris, 9.4% glycine and 0.5% SDS, pH 8.3). Protein samples were then boiled for 10 min, separated from the beads and transferred to new tubes.

### Immunoblot analysis

Individual protein samples were subjected to SDS-PAGE analysis, transferred to a polyvinylidene fluoride (PVDF) membrane (Roche), and blocked with freshly prepared blocking reagent (i.e. Tris-HCl buffer, pH 8.0, containing 0.05% Tween 20 and 3% skim milk) at 4°C for 12 h. Blocked membranes were incubated in 1:10,000 diluted mouse anti-FLAG tag monoclonal antibody or rabbit anti-HA tag polyclonal antibodies (α-HA; Proteintec, Wuhan) at 23°C for 1.5 h, and then washed five times with Tris-HCl buffer (pH 8.0) containing 0.08% NaCl and 0.05% Tween 20 (TBST). Subsequently, the membrane was incubated with HRP-conjugated affinity-purified goat-anti rabbit IgG (1: 10,000) at 23°C for 1 h, washed five times in TBST and reacted with 3,3′-diaminodbenzidine (Pierce, Thermo Fisher Scientific) to detect proteins.

### Structural modelling and docking

The three-dimensional structures of non-glycosylated *Tc*-MUC-1 (without a signal peptide) and its interacting proteins were modelled using the programs I-TASSER [38–40] and AlphaFold2 [41], and GO annotations predicted. *In silico* protein-protein docking was performed using the ClusPro server [42,43] based on resolved/modelled structures [44]. Protein complexes were analysed and displayed using the PyMOL molecular graphics system v2.5 (Schrödinger, LLC), and predicted binding sites in the *Tc*-MUC-1 and protein networks in murine macrophages.

## Results

### *Tc-*MUC-1 is predicted to have protein binding activity

**Fig 1:**
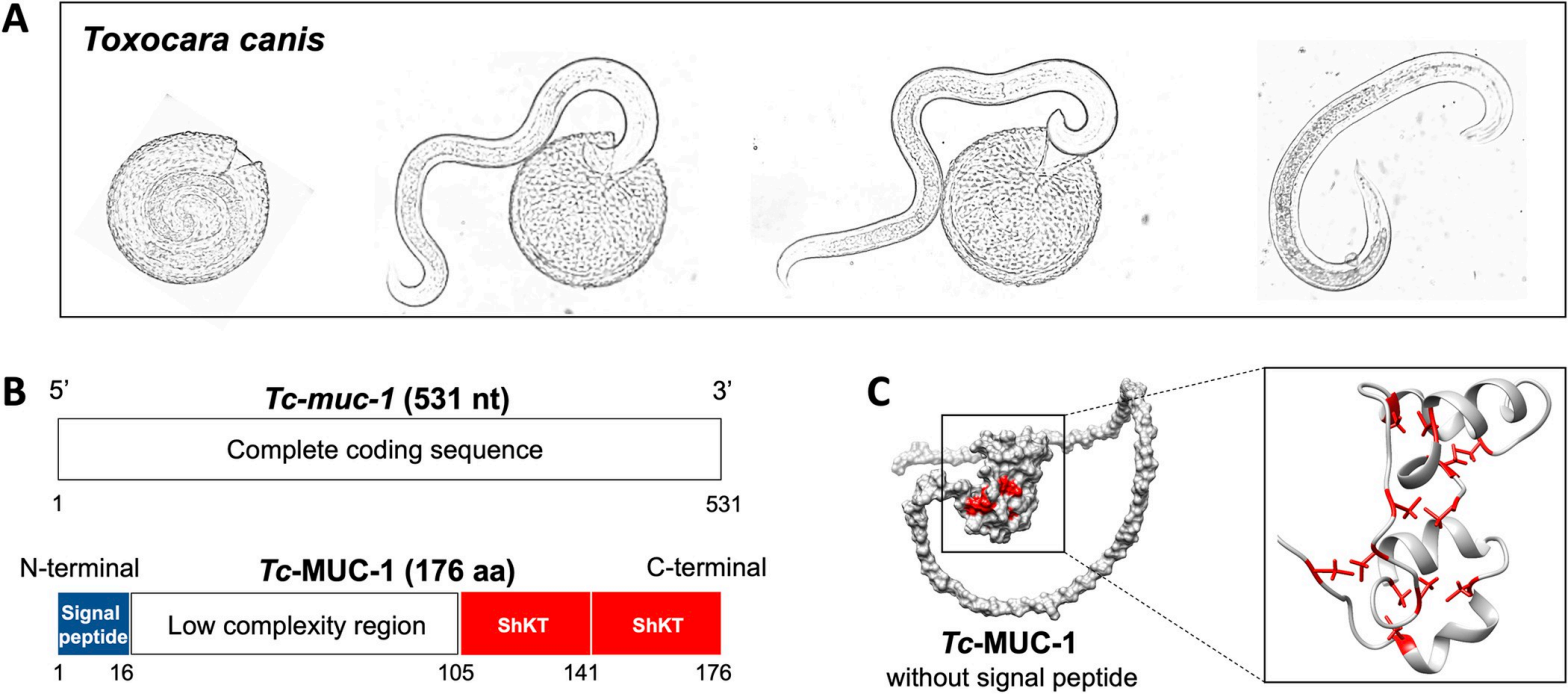
Molecular cloning and functional prediction of *Toxocara canis* mucin 1 (*Tc*-MUC-1). (**A**) Infective larvae of *T*. *canis* hatch from eggs *in vitro* are used for the molecular cloning of the gene (*Tc-muc-1*) encoding the *Tc*-MUC-1 protein. (**B**) Schematic diagram of *Tc-muc-1* and the predicted domain architecture of *Tc*-MUC-1. Signal peptide, low complexity region and two abutting ShKT domains are indicated. (**C**) The three-dimensional structure of *Tc*-MUC-1 (without signal peptide) modelled using AlphaFold2 [38]. Cysteine residues and disulfide bonds in each ShKT domain are indicated in red.

A complete cDNA sequence, called *Tc-muc-1* (531 nt in length), encoding the surface coat glycoprotein TES-120 precursor (*nmuc-1*) of infective larvae of *T*. *canis* (Fig 1A) was obtained. From this sequence, an amino acid sequence of 176 aa in length was deduced (called *Tc*-MUC-1) that contained a signal peptide, a low complexity region with STSSSS(P)A repeats, and two ShKT (also known as nematode six-cysteine domain or ion channel regulator) domains (Fig 1B). A U-shaped structure of *Tc*-MUC-1 was modelled, with two ShKT domains folded in the centre (Fig 1C). The fold of each ShKT domain contained two nearly perpendicular stretches of helices, stabilised by three disulfide bridges (Fig 1C). With reference to the functional information in the PDB database, ShKT domain-containing *Tc*-MUC-1 was predicted to have a binding activity (GO:0005488), particularly protein binding (GO:0005515) and metal ion binding (GO:0046872). No specific binding site was predicted with confidence, due to limited structural and functional information on mucins of *T*. *canis* and related parasitic worms.

### The r*Tc-*MUC-1 protein interacts with at least 219 proteins of murine macrophages

**Fig 2:**
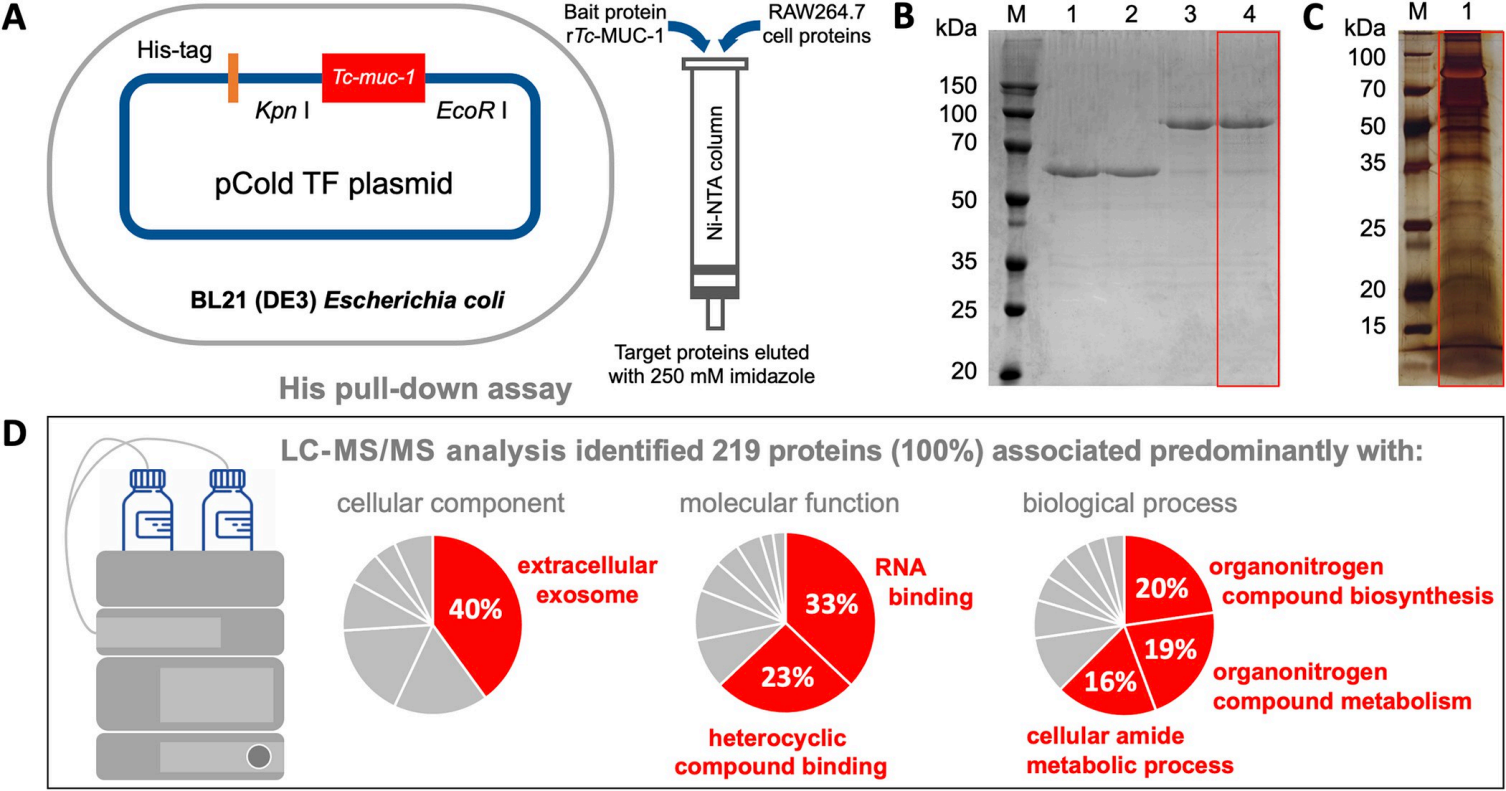
Pull-down and mass spectrometric analyses to identify proteins in murine macrophages that bind recombinant *Toxocara canis* mucin 1 (r*Tc*-MUC-1). (**A**) Prokaryotic expression of His-tagged bait protein (r*Tc*-MUC-1) and elution of proteins of RAW264.7 macrophages bound to a Ni-NTA column. (**B**) SDS-PAGE analysis of r*Tc*-MUC-1 and proteins pulled down by r*Tc*-MUC-1. M: protein marker; Lane 1: Empty His-tag peptide (~ 55 kDa); Lane 2: proteins of RAW264.7 cells targeted by the empty His-tag peptide; Lane 3: purified r*Tc*-MUC-1 fused to His-tag peptide (~ 75 kDa); Lane 4: proteins of RAW264.7 cells targeted by purified His-tag fused *rTc*-MUC-1 (red box indicated). (**C**) Proteins of RAW264.7 cells pulled down by r*Tc*-MUC-1, and resolved by SDS-PAGE and stained with silver stain (red box indicated). (**D**) LC-MS/MS analysis of proteins pulled down by r*Tc*-MUC-1. Peptides and proteins identified as well as the predominant functional predictions (cellular component, molecular function and biological processes) are indicated.

To understand aspects of the host-parasite interplay at the protein-protein level, molecules in murine RAW264.7 macrophage cells that directly or indirectly bound r*Tc*-MUC-1 of *T*. *canis* were analysed using His pull-down and subsequent mass spectrometry (Fig 2A–2D). A total of 618 peptides representing 219 proteins were identified by LC-MS/MS, 109 proteins for which at least two unique peptides (S2 and S3 Tables) were detected. Apart from 31 presently uncharacterised molecules, the other proteins identified (*n* = 188) were comprehensively annotated with Gene Ontology (GO) terms and/or linked to Kyoto Encyclopedia of Genes and Genomes (KEGG) pathways. The annotated proteins, predominantly associated with extracellular region and exosome, were functionally enriched in terms of RNA, protein, lipid and/or substrate binding, and are suggested to play roles in organonitrogen compound metabolism and biosynthetic processes (Figs 2D and S1 and S4 Table). These proteins appear to be involved particularly in multiple pathways, including metabolism, genetic information processing (e.g. protein processing in endoplasmic reticulum and RNA degradation), cellular processes (transport and catabolism, cellular community) and organismal systems (e.g. peroxisome, antigen processing and presentation pathway and nucleotide binding oligomerization domain (NOD)-like receptor signalling pathway) (S2 Fig and S5 Table). Based on information available for functional protein associations in the mouse, a complicated protein-protein interaction network was predicted for 147 of the mouse proteins identified (S3 Fig and S6 Table).

### The r*Tc-*MUC-1 protein binds a cofilin and a fatty acid binding protein of murine macrophages

**Fig 3:**
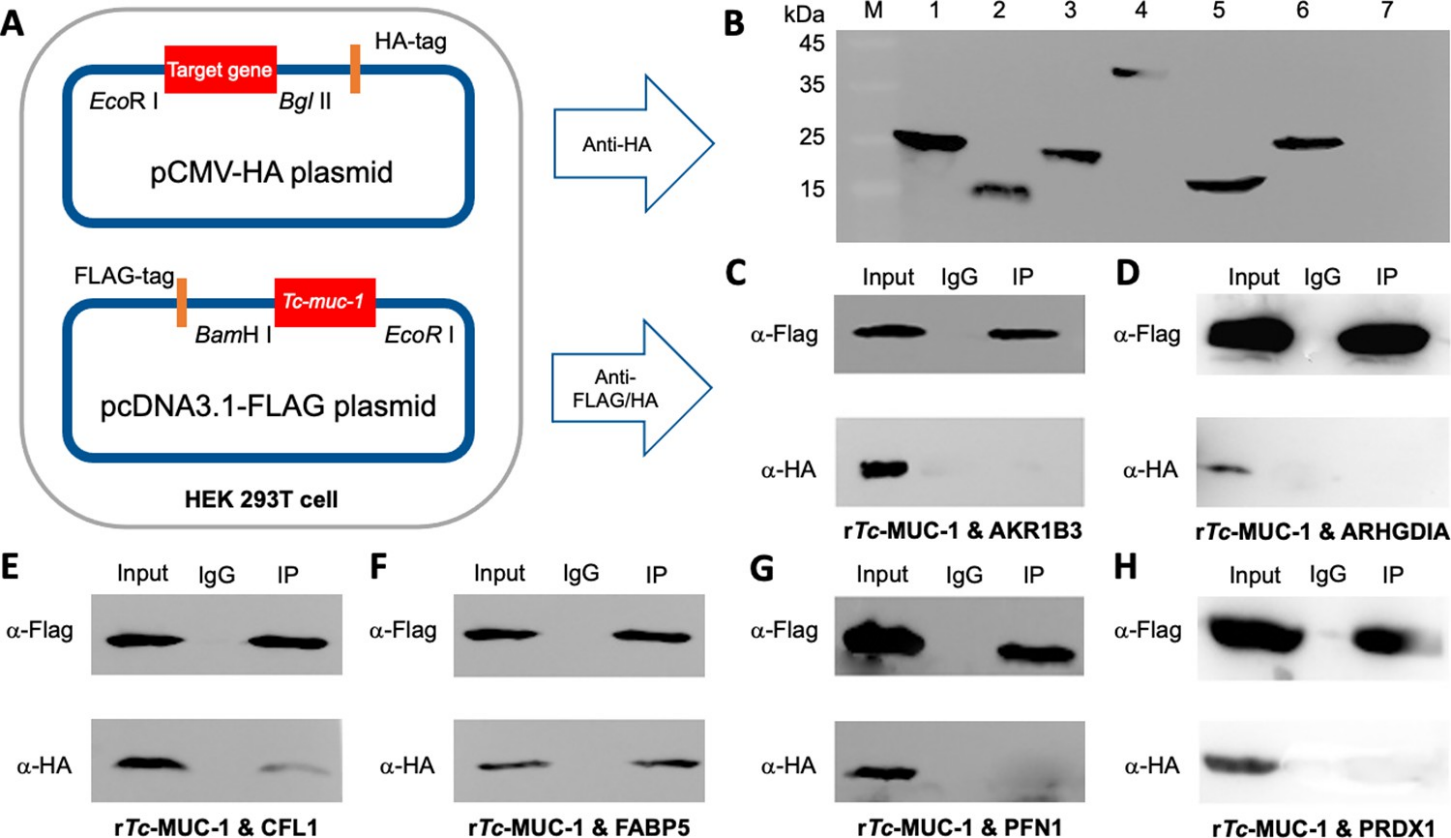
Co-immunoprecipitation analysis of proteins of murine macrophages bound to recombinant *Toxocara canis* mucin 1 (r*Tc*-MUC-1). (**A**) Eukaryotic expression of FLAG-tagged *Tc*-MUC-1 of *T*. *canis* and HA-tagged aldo_ket_red domain-containing protein (AKR1B3), rho GDP-dissociation inhibitor 1 (ARHGDIA), cofilin 1 (CFL1), fatty acid binding protein 5 (FABP5), peroxiredoxin 1 (PRDX1) and profilin (PFN1) of murine RAW264.7 macrophages in HEK 293T cells. (**B**) Western blot analyses of the expression of HA-tagged PRDX1 (lane 1), PFN1 (lane 2), CFL1 (lane 3), AKR1B3 (lane 4), FABP5 (lane 5), ARHGDIA (lane 6) and FLAG-tagged *Tc*-MUC-1 in transfected HEK 293T cells, using anti-haemagglutinin (HA)-tagged tag polyclonal antibodies (α-HA). M: Maker. Uncropped blot image can be found in S4 Fig. Western blot analyses of the expression of *Tc*-MUC-1, and (**C**) AKR1B3, (**D**) ARHGDIA, (**E**) CFL1, (**F**) FABP5, (**G**) PRDX1, and (**H**) PFN1 in transfected HEK 293T cells (Input), and in immunoprecipitated complex (IPC), using DYKDDDK tag monoclonal antibody (binds to FLAG tag epitope (α-Flag). Mouse IgG was used as a blank control.

The protein-protein interactions for selected molecules with enriched extracellular and functional annotations (S3 and S4 Tables) were examined in a human embryonic kidney cell line (HEK 293T). Selected candidates included aldo_ket_red domain-containing protein (AKR1B3; UniProt accession no. Q3UDY1), rho GDP-dissociation inhibitor 1 (ARHGDIA; UniProt accession no. Q99PT1), cofilin 1 (CFL1; UniProt accession no. Q544Y7), fatty acid binding protein 5 (FABP5; UniProt accession no. Q05816), peroxiredoxin 1 (PRDX1; UniProt accession no. P35700) and profilin (PFN1; UniProt accession no. Q8CEH8) of RAW264.7 cells (Unused score > 8; *P* value < 1.00E-40) (Fig 3A). HA-tagged candidates AKR1B3, ARHGDIA, CFL1, FABP5, PRDX1 and PFN1 as well as FLAG-tagged r*Tc-*MUC-1 were successfully expressed in HEK 293T cells, and identified by respective α-HA antibodies and α-FLAG antibodies (Fig 3B–3G). We found that only CFL1, FABP5, and potentially PFN1, of RAW264.7 murine macrophages were immunoprecipitated by r*Tc-*MUC-1 and recognised by both α-HA and α-FLAG antibodies (Fig 3E and 3F). No co-immunoprecipitation of r*Tc-*MUC-1 and recombinant AKR1B3, ARHGDIA or PRDX1 was evident (Fig 3C, 3D, 3G and 3H).

### Protein complexes with the r*Tc-*MUC-1 protein indicate non-covalent interactions

**Fig 4:**
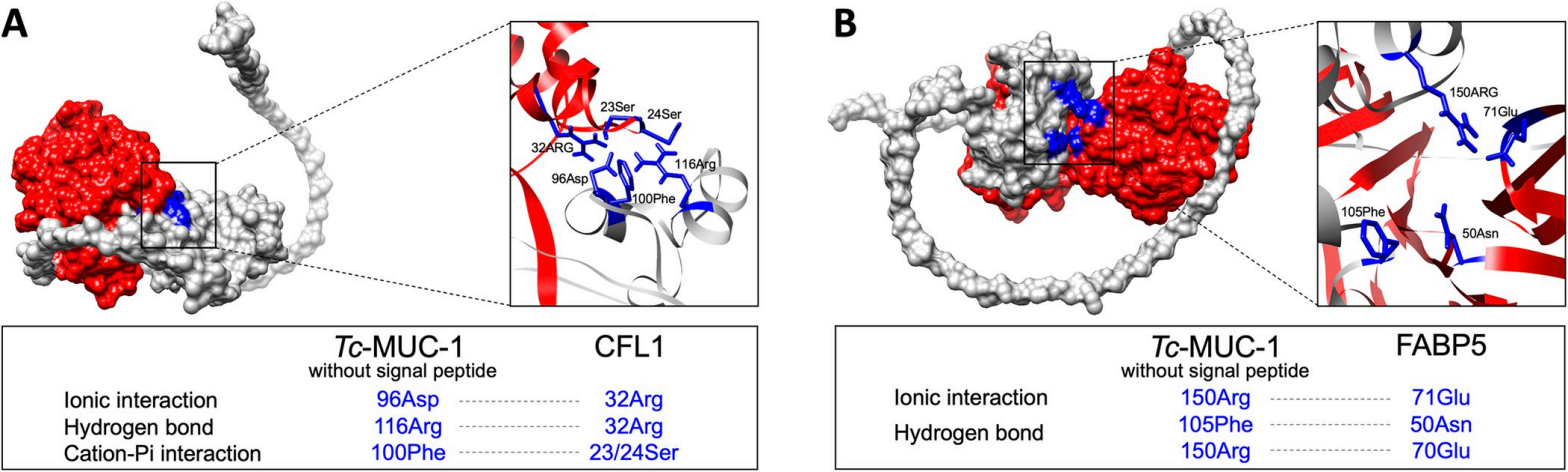
*In silico* docking of *Toxocara canis* mucin 1 (*Tc*-MUC-1) protein and two key interacting proteins. (**A**) Protein complex predicted for *Tc*-MUC-1 and the cofilin 1 (CFL1) of murine RAW264.7 macrophage cells. Structures of *Tc*-MUC-1 and CFL1 are indicated in grey and red, respectively. (**B**) Protein complex predicted for *Tc*-MUC-1 and a dimer of the fatty acid binding protein 5 (FABP5) of murine RAW264.7 macrophages. Structures of *Tc*-MUC-1 and FABP5 are indicated in grey and red, respectively. Ionic interactions, hydrogen bonds or cation-pi binding interaction between the residues in *Tc*-MUC-1 (grey) and CFL1 (red) are indicated.

Based on the experimentally confirmed physical interactions of r*Tc-*MUC-1 with CFL1 and FABP5, the corresponding protein complexes *Tc-*MUC-1–CFL1 and *Tc-*MUC-1–FABP5 were modelled (Fig 4). *Tc-*MUC-1–CFL1 was predicted to be stabilised by ionic interaction (between 96Asp in *Tc-*MUC-1 and 32Arg in CFL1), hydrogen bonds (between 116Arg in *Tc-*MUC-1 and 23/24Ser in CFL1), and cation-Pi binding interaction (between 100Phe in *Tc-*MUC-1 and 32Arg in CFL1) (Fig 4A). *Tc-*MUC-1–FABP5 was predicted to be stabilised by ionic interaction (between 150Arg in *Tc-*MUC-1 and 71Glu in FABP5) and hydrogen bonds (between 105 Phe and 150Arg in *Tc-*MUC-1 and 50Asn and 70Glu in FABP5) (Fig 4B). Although these protein-protein interactions were both non-covalent, different residues in *Tc-*MUC-1 are proposed to be involved and to bind multiple proteins.

### Interactions suggest that the *Tc-*MUC-1 protein is linked to cytoskeletal dynamics and signalling in macrophages

**Fig 5:**
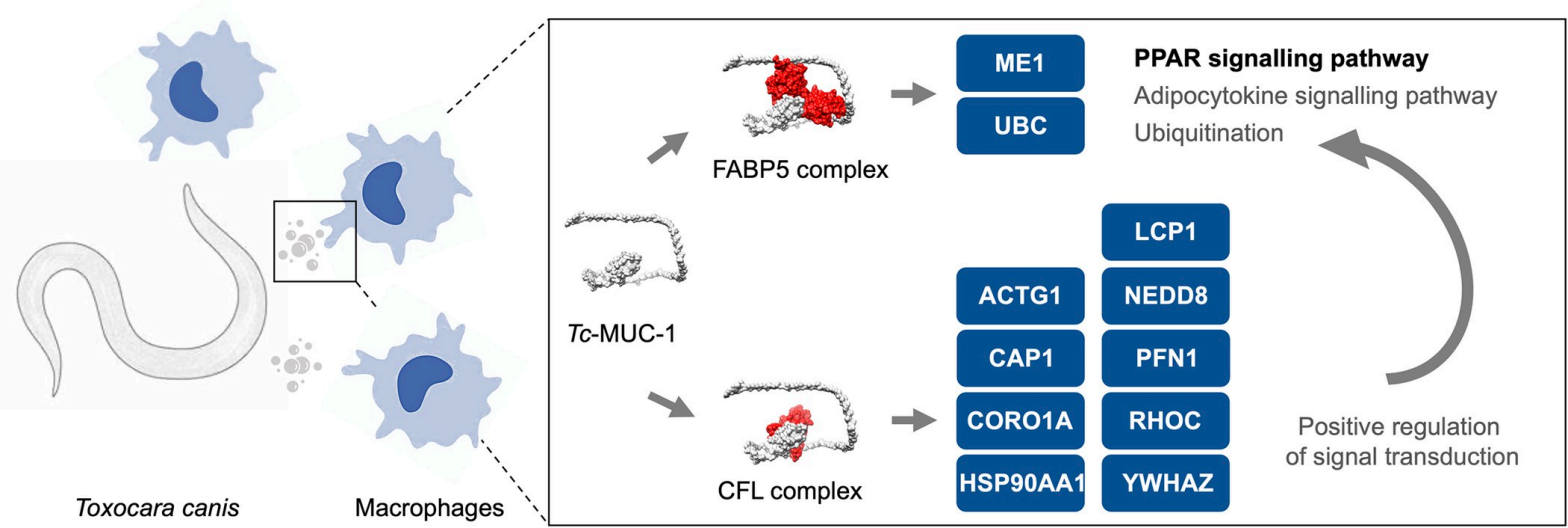
Schematic diagram summarising the inferred functions of cofilin 1 (CFL1) and fatty acid binding protein 5 (FABP5) that interact with a prokaryotically-expressed (non-glycosylated) recombinant form of the *Toxocara canis* mucin 1 protein (r*Tc*-MUC-1). FABP5 and CFL1 physically interact with r*Tc*-MUC-1, and are inferred to positively regulate cellular processes. FABP5 transports fatty acid ligands to activate peroxisome proliferator-activated receptors (PPAR), which then regulate the transcription of genes coding for malic enzyme 1 (ME1) and for ubiquitin C (UBC), participating in adipocytokine signalling pathway associated lipogenesis and ubiquitination, respectively. CFL interacts with actin gamma 1 (ACTG1), cyclase associated actin cytoskeleton regulatory protein 1 (CAP1), coronin actin binding protein 1A (CORO1A), heat shock protein 90 alpha family class A member 1 (HSP90AA1), lymphocyte cytosolic protein 1 (LCP1), ubiquitin-like modifier (NEDD8), PFN1, Ras homologue family member C (RHOC) and 14-3-3 protein zeta (YWHAZ), to positively regulate ME1-associated signal transduction in the adipocytokine signalling pathway.

Focusing on the r*Tc*-MUC-1–CFL1 and r*Tc*-MUC-1–FABP5 interactions, functional information on other proteins from murine macrophages initially identified in the pull-down assay were manually curated. CFL1 has a relatively complicated network with 17 of the identified proteins of RAW264.7 cells (S6 Table), including ARHGDIA and PFN1, which were not, or only weakly, identified using the co-immunoprecipitation (co-IP) assay. These molecules appear to dominate functionally in cell activation (e.g. cellular response to stimulus) and actin filament-based processes (e.g. extracellular matrix organization, cell migration and adhesion), and in actin-based cytoskeletal dynamics regulation-associated signalling (S7 and S8 Tables). Although no protein association network was predicted for FABP5 (S6 Table), this fatty acid-binding protein as well as malic enzyme 1 (ME1) and ubiquitin C (UBC) were predicted to be involved in the peroxisome proliferator-activated receptor (PPAR) signalling pathway (S8 Table). An integration of the biological information for CFL1, FABP5 and associated proteins indicates roles for *Tc-*MUC-1 in regulating the cytoskeletal dynamics and adipocytokine signalling of murine macrophages, particularly in the positive regulation of extracellular matrix organization (Fig 5).

## Discussion

Using an integrated pull-down, mass spectrometry, co-IP and bioinformatic approach, we investigated the interactions between the non-glycosylated form of the *Tc-*MUC-1 protein and macrophages of the murine host; r*Tc*-MUC-1 was shown to be physically interactive with CFL1 and FABP5 from murine RAW264.7 macrophage cells. The integration of information from the protein association networks/pathways for the two proteins suggested that the *Tc*-MUC-1 protein plays a role in interfering cytoskeletal organisation and signal transduction in host macrophages.

The surface coat glycoprotein MUC-1 of *T*. *canis* has a protein binding activity. In the 1990s, it was shown that the mucin- and glycan-rich assembly form the “fuzzy coat” of *T*. *canis*, which appears to play a key role in shedding the surface coat with bound immune cells [20,45,46]. To date, a family of secreted mucins, namely MUC-1, -2, -3, -4 and -5, has been identified in the infective larvae of *T*. *canis* [47,48]. The proteins of each of these molecules are “hybrids”, being unique among mucins in having acquired two ShKT domains [47,48]. One possibility is that this acquisition has conferred new properties, such as the interaction with proteins of host immune cells that promote infection. Although the ShKT domain is found in the potassium channel inhibitor ShK (a toxin) in sea anemone [49], it has been proposed to bind molecules without exhibiting a toxic effect [47]. Indeed, by performing His pull-down and co-IP assays, we confirmed that r*Tc*-MUC-1 did bind to the actin binding protein CFL1 of macrophages and a fatty acid binding protein FABP5 of the RAW264.7 cell line. The *Tc-*MUC-1–CFL1 and *Tc-*MUC-1–FABP5 complexes were modelled *in silico*, and strong hydrogen bonds were predicted, supporting previous speculation that the formation of intermolecular complexes with the ShK/SXC domains might be through non-covalent means [47]. Crystal structures of these complexes should confirm the binding sites of the *Tc*-MUC-1 protein, although protein structures were modelled with the machine learning-based program AlphaFold2 [41]. N-glycosylation of *Tc*-MUC-1-FLAG in HEK 293T cells might explain why no interactions with AKR1B3, ARHGDIA, PFN1 or PRDX1 that were detected by pull-down experiment. Future mutation experiments should define which key residues in r*Tc*-MUC-1 are directly involved in binding CFL1 and/or FABP5 of murine RAW264.7 macrophages.

By binding to the actin binding protein cofilin 1, the *Tc*-MUC-1 protein is likely to interfere with the migration and phagocytosis of macrophage cells. Like other pathogen molecules detected by host animals, the mucins of parasites can be recognised by innate immune cells [20,50]. It is well known that the specialised monocytes (e.g. macrophages) play essential roles in the detection and elimination of pathogens, including parasitic worms [51–53]. In this process, cell migration and phagocytosis are involved, which rely on a dynamic remodelling of actin cytoskeleton. Cofilin is one of the actin-binding proteins demonstrated to play roles in the actin cytoskeletal remodelling, migration directionality and phagosome formation of macrophages [54,55]. Interestingly, in the current study, we showed that the r*Tc*-MUC-1 interacts with the actin binding protein CFL1 of the macrophage-like Abelson leukaemia virus-transformed cell line derived from BALB/c mice (RAW264.7) that has been commonly used to explore parasite-host interactions [56–58]. CFL1 was also predicted to associate with other proteins that were identified in the pull-down assay, including actin, cyclase associated actin cytoskeleton regulatory protein, coronin actin binding protein, lymphocyte cytosolic protein and Rho GDP dissociation inhibitor alpha, which are known to play roles in the myosin contraction and cofilin-mediated disassembly during macrophage migration [54,55]. These results strongly suggest that the *Tc*-MUC-1 protein, by interacting with CFL1, affects the migration and phagosome formation of host macrophages in innate immune responses.

The *Tc*-MUC-1 protein might also play a role in regulating the signal transduction in host immune cells. This statement could be supported by an association network of CFL1 with the identified signalling molecules involved in cell shape, attachment and motility, including Rho GTPases and 14-3-3 protein zeta [59–61]. Therefore, by interacting with CFL1, the *Tc*-MUC-1 protein is likely to influence the cell locomotion of stimulated macrophages; this protein might also play a role in modulating inflammation-associated signalling pathways, as interacting molecules (i.e. fatty acid binding protein 5 and the associated NADP-dependent enzyme ME1 and polyubiquitin precursor UBC) are known to transfer specific fatty acids from the cytosol to the nucleus, wherein they activate nuclear receptors linked to the modulation of NF-kappa-B signalling during inflammation [62,63]. Indeed, mucins of *T*. *canis* have been reported to play roles in the stimulation of immune cells of host animals, particularly via IL-10 and proinflammation cytokine production and Toll-like receptor signalling *in vitro* [23,24]. Further studies should explore the regulatory role(s) of *Tc*-MUC-1 on immune recognition and/or evasion (e.g., *via* macrophage proliferation/activation, expression of major histocompatibility complex (MHC) class II molecules, cytokine secretion and/or calcium flux).

Questions surrounding *Toxocara* mucins that also warrant investigation in the future include: Do sugar moieties of *Tc*-MUC-1 have an impact on its recognition by host macrophage cells?–as it has been reported that such elements are important in antibody-mediated immune responses [27]. Do the ShKT domains of the *Tc*-MUC-1 protein play roles in altering innate immune responses of host animals?–as ShKT domain-containing peroxidase and associated cuticle development of *C*. *elegans* has been reported to be involved in pathogen resistance [64–66]. Are mucins involved in the tuft cell activation?–which has been emerging a novel aspect for understanding intestinal immune responses to parasitic infections [67]. Addressing these questions should improve our understanding of the interplay between *T*. *canis* larvae and host cells.

In conclusion, we demonstrated that a non-glycosylated, recombinant form of the protein of *Tc*-MUC-1 interacts with the actin binding protein CFL1 and the fatty acid binding protein FABP5 of murine RAW264.7 macrophages *in vitro*. These findings suggest that the *Tc*-MUC-1 protein might interfere with the migration and phagocytosis of host macrophages, providing new insight into the host-parasite interplay at the molecular and cellular levels. Further investigations of glycosylated *Tc*-MUC-1 in the interaction with host (murine and human) macrophages, and other species of *Toxocara* and ascaridoids should establish whether immune recognition and modulation are genus- or species-specific.

## Supporting information

S1 Table Oligonucleotide primer sets used for the PCR-based amplification of coding sequences for subsequent protein expression in HEK 293T cells. (XLSX)

S2 Table Peptides of proteins of RAW264.7 cells identified to interact with the r*Tc*-MUC-1 protein by His pull-down and LC-MS/MS. (XLSX)

S3 Table Proteins of RAW264.7 cells identified to interact with the r*Tc*-MUC-1 protein by His pull-down and LC-MS/MS. (XLSX)

S4 Table Gene Ontology (GO) annotation of proteins of RAW264.7 cells identified to interact with the r*Tc*-MUC-1 protein. (XLSX)

S5 Table Kyoto Encyclopedia of Genes and Genomes (KEGG) annotation of proteins of RAW264.7 cells identified to interact with the r*Tc*-MUC-1 protein. (XLSX)

S6 Table Networks of proteins of RAW264.7 cells identified to interact with the r*Tc*-MUC-1 protein. (XLSX)

S7 Table Biological process annotations for proteins of RAW264.7 cells identified to interact with CFL1 and FABP5. (XLSX)

S8 Table Pathway annotations of proteins of RAW264.7 cells identified to interact with CFL1 and FABP5. (XLSX)

S1 Fig Gene Ontology (GO) annotation of proteins of RAW264.7 cells identified to interact with the r*Tc*-MUC-1 protein. Enrichment analyses of proteins bound to recombinant *Toxocara canis* mucin 1 (r*Tc*-MUC-1) for (**A**) cellular component, (**B**) molecular function, and (**C**) biological process. Predominant annotation enrichments and the percentages are underlined.
(TIF)

S2 Fig Pathway enrichment for proteins of RAW264.7 cells identified to interact with the r*Tc*-MUC-1 protein. (TIF)

S3 Fig Protein association network predicted for the proteins identified in RAW264.7 cells. (TIF)

S4 Fig Western blot analyses of the expression of HA-tagged proteins of RAW264.3 and FLAG-tagged *Tc*-MUC-1 in transfected HEK 293T cells. Lanes 1–7: PRDX1, PFN1, CFL1, AKR1B3, FABP5, ARHGDIA and FLAG-tagged *Tc*-MUC-1. M: Maker.
(TIF)
