# Supplementary figures and images for: The non-glycosylated protein of Toxocara canis MUC-1 interacts with proteins of murine macrophages
Source: PLoS Negl Trop Dis. 2022 Sep 2;16(9):e0010734. doi: 10.1371/journal.pntd.0010734 (PMC9477421; doi:10.1371/journal.pntd.0010734)

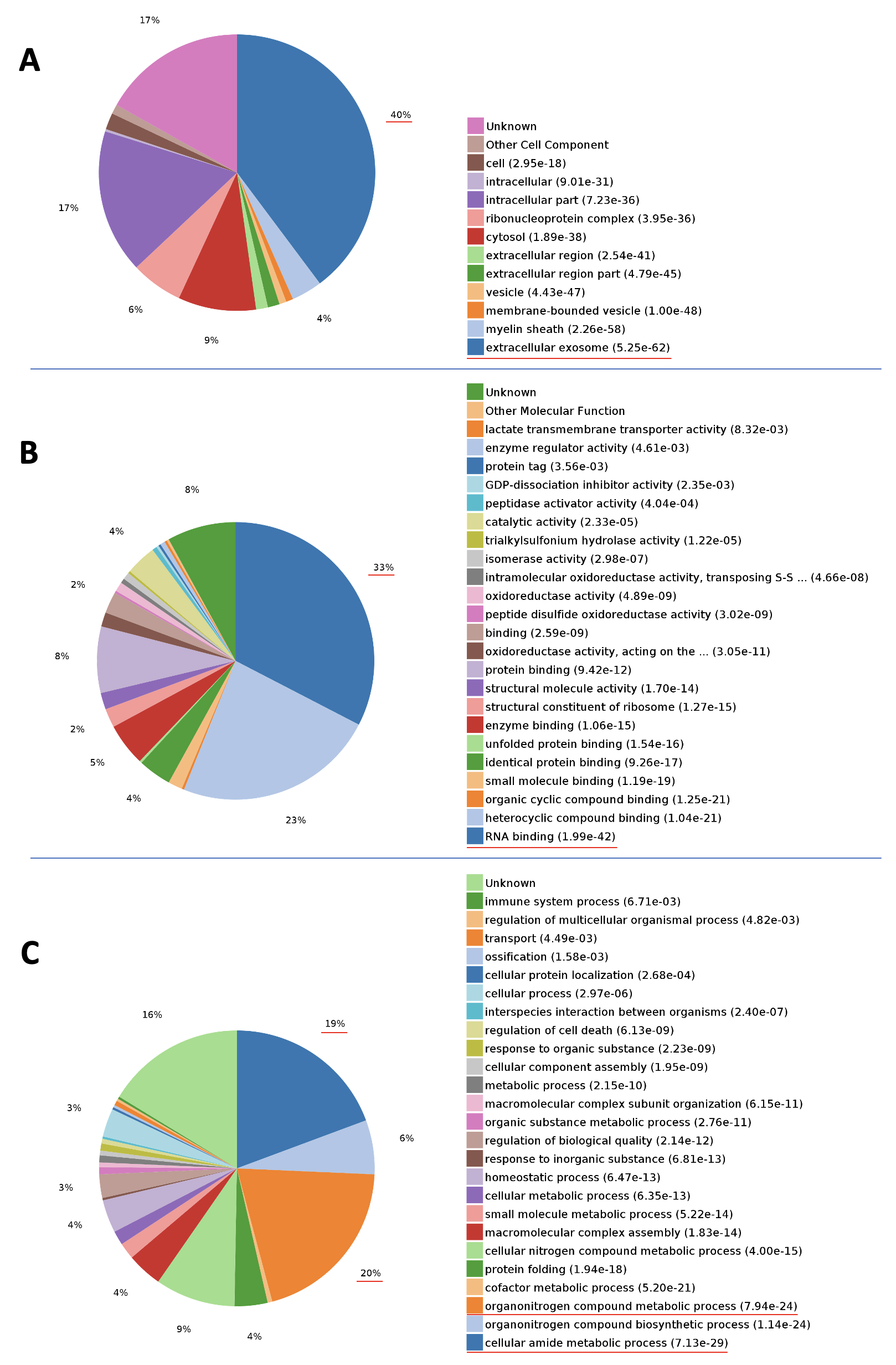

Supplement: S1 Fig — Enrichment analyses of proteins bound to recombinant Toxocara canis mucin 1 (rTc-MUC-1) for (A) cellular component, (B) molecular function, and (C) biological process. Predominant annotation enrichments and the percentages are underlined. (TIF) [file pntd.0010734.s009.tif]

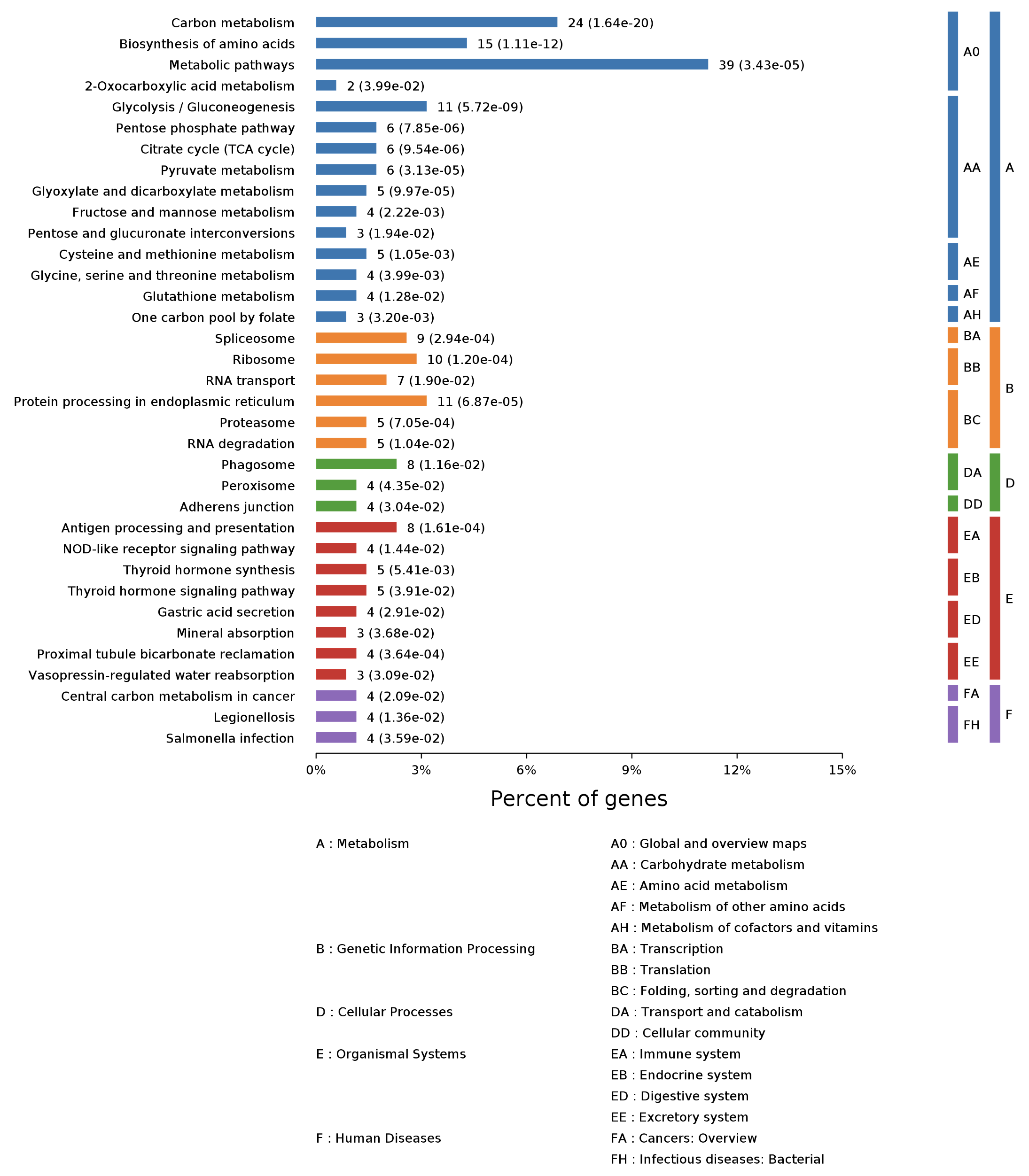

Supplement: S2 Fig — (TIF) [file pntd.0010734.s010.tif]

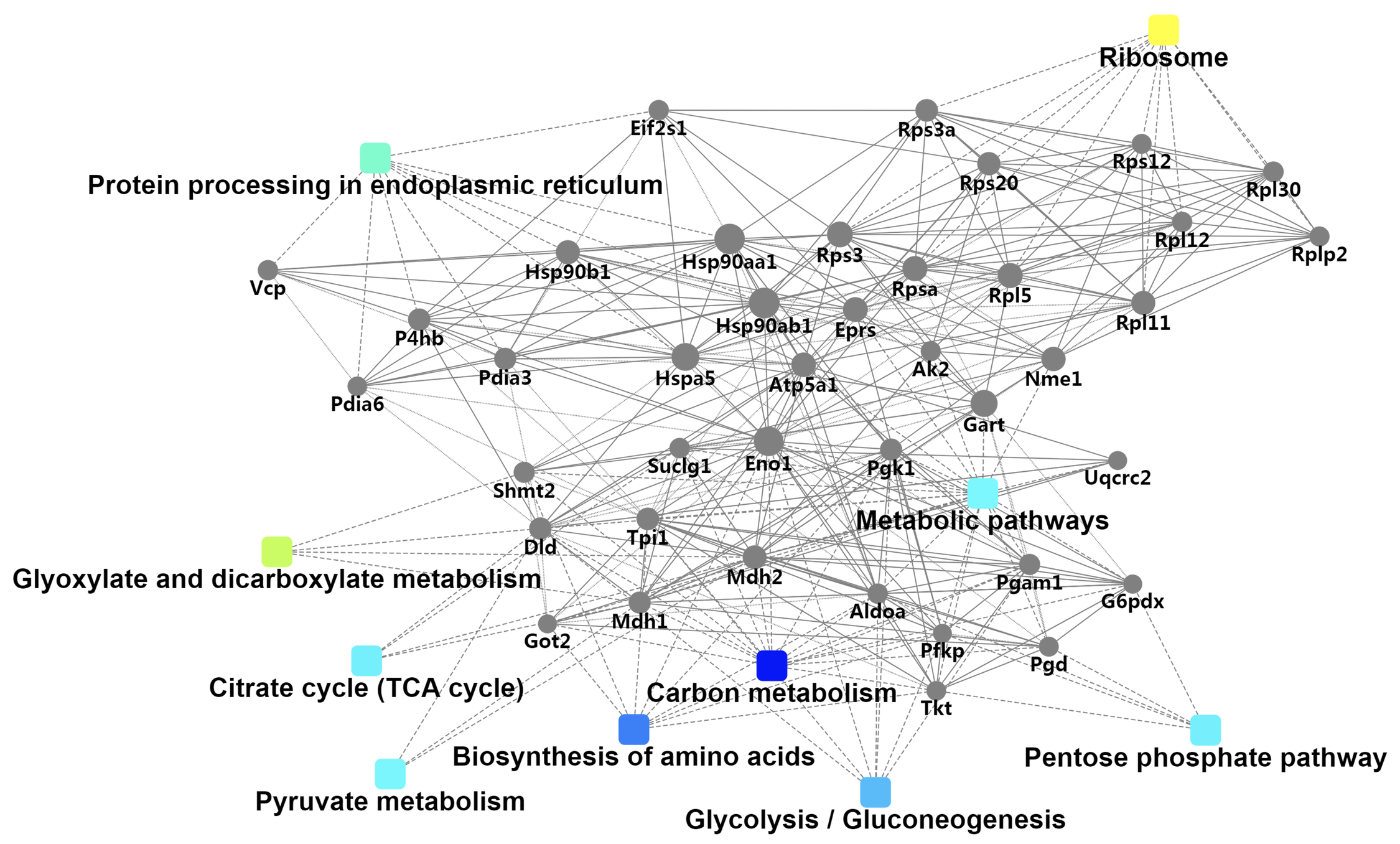

Supplement: S3 Fig — (TIF) [file pntd.0010734.s011.tif]

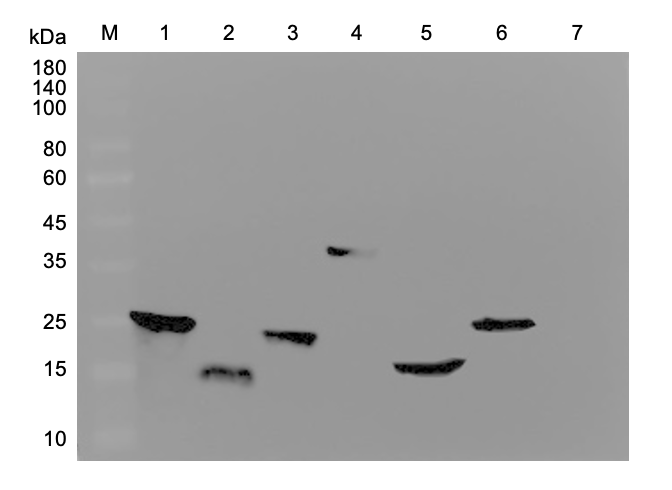

Supplement: S4 Fig — Lanes 1–7: PRDX1, PFN1, CFL1, AKR1B3, FABP5, ARHGDIA and FLAG-tagged Tc-MUC-1. M: Maker. (TIF) [file pntd.0010734.s012.tif]
